# Wearable Sensors for Human Health Monitoring and Analysis

**DOI:** 10.3390/s26020575

**Published:** 2026-01-15

**Authors:** Alessandro Scano, Rebecca Re, Paolo Perego, Alfonso Mastropietro

**Affiliations:** 1 Laboratory of Advanced Methods for Biomedical Signal and Image Processing, Institute of Intelligent Industrial Technologies and Systems for Advanced Manufacturing (STIIMA), Italian National Research Council (CNR), 20133 Milan, Italy; alfonso.mastropietro@cnr.it; 2 Department of Physics of Politecnico di Milano, Institute of Photonics and Nanotechnologies, Italian National Research Council (CNR), 20133 Milan, Italy; rebecca.re@polimi.it; 3 Department of Design, Politecnico di Milano, Via Candiani 72, 20158 Milan, Italy; paolo.perego@polimi.it

## 1. Introduction

Many fields of research have recently made significant progress with the use of wearable sensors for the evaluation, monitoring, and analysis of health in several contexts, including healthcare [1,2], rehabilitation [3], industry [4], sports [5], and others [6]. Such sensors foster human-centered approaches to research and innovative applications and extend practical use, playing key roles in improving people’s lives [7]. However, the adoption of wearable sensors poses several scientific challenges, including issues regarding data management, effective data analysis, calibration, the design of novel sensors (regarding wearability and power supply issues), and novel sensors’ adherence to previously adopted standards. There are even logistical challenges to face, related, for example, to compatibility with and adherence to mandatory guidelines, especially in critical scenarios (such as those in medicine) [8].

In this Special Issue, “Wearable Sensors for Human Health Monitoring and Analysis,” we aimed to foster scientific interest in these challenges and gathered a collection of 12 papers that describe the diverse applications of wearable biomedical sensors in the healthcare context pertaining to wearable sensors for human health monitoring and analysis. These contributions are grouped into key thematic areas, reflecting the breadth of research:

1. *Neurophysiology and brain signal analysis*. The authors of papers in this category utilized neurophysiological techniques such as electroencephalography (EEG) and functional near-infrared spectroscopy (fNIRS) to evaluate cerebral activity, pain intensity, and movement control, including studies on multilevel pain assessment using fNIRS and design suggestions for the optimal way of using wearable EEG equipment to evaluate motor imagery.
2. *Muscle and neuromotor analysis through physiological sensors*. This area of research investigates the monitoring or classification of neuromuscular and motor activity (EMG, sEMG, and muscle synergies), with applications in clinical, biomechanical, and sports contexts. It includes electromyographic comparisons of training equipment and multi-scale CNNs for sEMG-based hand gesture recognition for prosthetic devices.
3. *Wearable motion sensors and human motion evaluation*. These papers focus on the design, validation, and calibration of wearable sensors to analyze complex movements among frail individuals or those performing physical activities. Notable contributions address analyzing optimal wearable motion sensor placement for accurate classification of fall directions and the validity and test–retest reliability of spatiotemporal running parameter measurement. The utility of calibrating wearable sensors for quantifying infant leg movements is also explored.
4. *Design of and technologies used for wearable devices and digital health*. The studies in this category explore the design and development of novel hardware and software solutions for wearable systems, with emphasis on efficiency, energy, and data management. It includes studies on hybrid energy-efficient harvesting systems for healthcare wearables as well as the design and development of a smart fidget toy using blockchain technology to improve health data control.

Taken together, these papers highlight the key role of wearable sensors in understanding physiological mechanisms and advancing healthcare, medicine, industry, and sports. They further promote the adoption of these devices by providing insights for use, practical implementation strategies, software and hardware design considerations, and novel algorithms. A complete list of the contributors to this Special Issue is provided below:

- Zhu, W.; Lin, Y. Physiological Sensor Modality Sensitivity Test for Pain Intensity Classification in Quantitative Sensory Testing. Sensors 2025, 25, 2086. https://doi.org/10.3390/s25072086.
- Falivene, A.; Johnson, C.; Klingels, K.; Meyns, P.; Verbecque, E.; Hallemans, A.; Biffi, E.; Piazza, C.; Crippa, A. Time-Normalization Approach for fNIRS Data During Tasks with High Variability in Duration. Sensors 2025, 25, 1768. https://doi.org/10.3390/s25061768.
- Abelleira-Lamela, T.; Marcos-Pardo, P.J.; Abraldes, J.A.; González-Gálvez, N.; Espeso-García, A.; Esparza-Ros, F.; Vaquero-Cristóbal, R. Electromyographic Comparison of Traditional Fitness Machines, Outdoor Fitness Equipment Without Load Selectors, and Outdoor Fitness Equipment with Load Selectors in a Seated Chest Press Exercise in Trained Young Men. Sensors 2024, 24, 7740. https://doi.org/10.3390/s24237740.
- Fratti, R.; Marini, N.; Atzori, M.; Müller, H.; Tiengo, C.; Bassetto, F. A Multi-Scale CNN for Transfer Learning in sEMG-Based Hand Gesture Recognition for Prosthetic Devices. Sensors 2024, 24, 7147. https://doi.org/10.3390/s24227147.
- Bobrova, P.; Perego, P.; Boiano, R. Design and Development of a Smart Fidget Toy Using Blockchain Technology to Improve Health Data Control. Sensors 2024, 24, 6582. https://doi.org/10.3390/s24206582.
- Teng, S.; Kim, J.-Y.; Jeon, S.; Gil, H.-W.; Lyu, J.; Chung, E.H.; Kim, K.S.; Nam, Y. Analyzing Optimal Wearable Motion Sensor Placement for Accurate Classification of Fall Directions. Sensors 2024, 24, 6432. https://doi.org/10.3390/s24196432.
- Riglet, L.; Orliac, B.; Delphin, C.; Leonard, A.; Eby, N.; Ornetti, P.; Laroche, D.; Gueugnon, M. Validity and Test–Retest Reliability of Spatiotemporal Running Parameter Measurement Using Embedded Inertial Measurement Unit Insoles. Sensors 2024, 24, 5435. https://doi.org/10.3390/s24165435.
- Tohidinejad, Z.; Danyali, S.; Valizadeh, M.; Seepold, R.; TaheriNejad, N.; Haghi, M. Designing a Hybrid Energy-Efficient Harvesting System for Head- or Wrist-Worn Healthcare Wearable Devices. Sensors 2024, 24, 5219. https://doi.org/10.3390/s24165219.
- Carretero, A.; Araujo, A. Design Decisions for Wearable EEG to Detect Motor Imagery Movements. Sensors 2024, 24, 4763. https://doi.org/10.3390/s24154763.
- Khan, M.U.; Sousani, M.; Hirachan, N.; Joseph, C.; Ghahramani, M.; Chetty, G.; Goecke, R.; Fernandez-Rojas, R. Multilevel Pain Assessment with Functional Near-Infrared Spectroscopy: Evaluating ΔHBO2 and ΔHHB Measures for Comprehensive Analysis. Sensors 2024, 24, 458. https://doi.org/10.3390/s24020458.
- Oh, J.; Loeb, G.E.; Smith, B.A. The Utility of Calibrating Wearable Sensors before Quantifying Infant Leg Movements. Sensors 2024, 24, 5736. https://doi.org/10.3390/s24175736.
- Scano, A.; Lanzani, V.; Brambilla, C.; d’Avella, A. Transferring Sensor-Based Assessments to Clinical Practice: The Case of Muscle Synergies. Sensors 2024, 24, 3934. https://doi.org/10.3390/s24123934.

A brief description of the novel contributions and main challenges faced in this Special Issue is provided in the following section.

## 2. Neurophysiology and Brain Signal Analysis

This section focuses on neurophysiology and brain signal analysis, sharing as a common topic the use of non-invasive neurophysiological techniques (EEG and fNIRS) to evaluate cerebral activity, pain intensity, or movement control. 

“Time-Normalization Approach for fNIRS Data During Tasks with High Variability in Duration” presents a novel algorithm that aids in the comparison of multiple acquisitions during (fNIRS) recordings. The proposed algorithm performed well on a set of experimental data presented by the authors; it was distributed with the publication and has been made available to users.

“Multilevel Pain Assessment with Functional Near-Infrared Spectroscopy: Evaluating ΔHBO2 and ΔHHB Measures for Comprehensive Analysis” assesses pain in non-verbal patients. The study focuses on assessing pain via the analysis of fNIRS signals combined with machine learning, utilizing multiple fNIRS measures, including oxygenated and deoxygenated hemoglobin. The combination of these two measures demonstrated superior performance relative to when they were used independently in multilevel pain analysis.

Finally, “Design Decisions for Wearable EEG to Detect Motor Imagery Movements” investigated how one can make informed decisions regarding the design of wearable electroencephalography (wearable EEG) devices for the evaluation of motor imagery, drawing several conclusions regarding the optimal sampling frequency, the EEG system’s comfort and portability, trials, the number of electrodes, algorithms, and their parameters, thus providing a set of practical suggestions for experimenters.

## 3. Muscle and Neuromotor Analysis Through Physiological Sensors

This section focuses on muscle and neuromotor analysis via physiological sensors, investigating the monitoring and classification of neuromuscular or motor activities (EMG, sEMG, muscle synergies, and movement) with clinical, biomechanical, or sports-related aims.

“Physiological Sensor Modality Sensitivity Test for Pain Intensity Classification in Quantitative Sensory Testing” deals with chronic pain and introduces a novel framework for objectively classifying pain intensity levels using physiological signals (blood volume pulse (BVP), galvanic skin response, EMG, respiration rate, skin temperature, and pupillometry) during Quantitative Sensory Testing sessions based on multiple wearable sensors, revealing that BVP is the most critical one for understanding pain levels.

Furthermore, “Electromyographic Comparison of Traditional Fitness Machines, Outdoor Fitness Equipment Without Load Selectors, and Outdoor Fitness Equipment with Load Selectors in a Seated Chest Press Exercise in Trained Young Men” describes an EMG assessment of the upper limbs of young men during their use of outdoor fitness equipment. The authors concluded that training with the outdoor seated chest press generated less EMG activity than traditional machine training. However, in general, an outdoor chest press equipped with a load selector system proved effective for strength training, yielding results comparable to those achieved the seated chest press.

“A Multi-Scale CNN for Transfer Learning in sEMG-Based Hand Gesture Recognition for Prosthetic Devices” describes how advancements in neural network approaches have enhanced the effectiveness of surface electromyography (sEMG)-based hand gesture recognition when measuring muscle activity. However, such methods hardly achieve high generalization and robustness, often requiring significant computational resources. In developing a robust model that can quickly adapt to new datasets using transfer learning, the authors highlight the effectiveness of transfer learning in creating adaptive, user-specific models for sEMG-based prosthetic hands.

Finally, “Transferring Sensor-Based Assessments to Clinical Practice: The Case of Muscle Synergies” expands on how advanced methods for analyzing motor control (muscle synergies) can be used to enhance the comprehension of the neuromotor system and adapt and predict the outcomes of therapies conceived to restore motor function. The paper also discusses the practical and technical challenges to be faced in adopting muscle synergies in clinical environments in research and in clinical practice.

## 4. Wearable Motion Sensors and Human Motion Evaluation

This section includes research that discusses wearable motion sensors and human motion evaluation, aiming at designing, validating, and calibrating wearable sensors to analyze complex movements or physical activity.

The authors of “Analyzing Optimal Wearable Motion Sensor Placement for Accurate Classification of Fall Directions” assessed inertial measurement unit (IMU) sensors placed at 12 distinct body locations to determine the most effective positions for capturing fall-related data. Statistical analyses of the results for the most effective classifier model demonstrated that the support vector machine is more effective than other classifiers across all sensor locations, with statistically significant differences in performance; the optimal sensor configuration for fall-direction classification involves strategically combining sensors placed on the pelvis, upper legs, and lower legs.

Furthermore, “Validity and Test–Retest Reliability of Spatiotemporal Running Parameter Measurement Using Embedded Inertial Measurement Unit Insoles” discusses the validation of the DSPro^®^ insoles, developed to collect running parameters during tasks. The aim of this study was to assess the test–retest reliability and criterion validity of running gait parameters from DSPro^®^ insoles compared to a motion-capture system. The test–retest reliability reflected moderate to excellent ICC values (ICC > 0.50).

Finally, in “The Utility of Calibrating Wearable Sensors before Quantifying Infant Leg Movements”, the authors used wearable sensors to measure infant leg movement, showing that offset error in the measurement of gravitational acceleration is common among commercially available sensors. They demonstrate how offset and other errors can be measured using three wearable sensors available to professionals and how they affected a threshold-based movement detection algorithm for the quantification of infant leg movement. Additionally, they reveal how these offsets can be calibrated and corrected for to increase the reproducibility of results across sensors.

## 5. Design and Technologies for Wearable Devices and Digital Health

This section includes papers that describe design and technologies pertaining to wearable devices and digital health, focusing on the development of hardware/software wearable systems, with attention to efficiency, energy, and data management.

In “Designing a Hybrid Energy-Efficient Harvesting System for Head- or Wrist-Worn Healthcare Wearable Devices,” the authors describe the architecture of a system based on highly efficient photovoltaic panels, compact thermoelectric modules, and two ultra-low-power BQ25504 DC-DC boost converters (Texas Instruments, Dallas, United States) which can increase battery life from 9.31 h to over 18 h, making it suitable for health-monitoring wearables worn on the head, face, or wrist region, specifically for outdoor workers.

“Design and Development of a Smart Fidget Toy Using Blockchain Technology to Improve Health Data Control” explores the integration of blockchain technology in wearable health devices through the design and development of a SmartFidgetToy. Using an iterative user-centered design approach, the authors developed a mid-fidelity prototype of a physical fidget device with a blockchain-based web application. The study revealed high user interest (70%) in blockchain-based data control and sharing features and improved perceived security of data (among 90% of users) with blockchain integration.

## 6. Conclusions

This Special Issue, “Wearable Sensors for Human Health Monitoring and Analysis,” presents a comprehensive collection of cutting-edge research in the field of wearable sensors. The 12 papers featured in the collection emphasize the rapid evolution and interdisciplinary nature of this field, offering significant contributions to both fundamental understanding and practical applications in human-health monitoring. Spanning novel approaches to pain *assessment using* fNIRS and sEMG-based prosthetic device control, advancements in fall detection and energy-efficient power solutions, and the pioneering use of blockchain for health data management, the collection highlights the enormous potential of wearable technology in healthcare. These advancements in the use of wearable technologies pave the way for improved practices, novel design approaches and paradigms, and advanced research programs. As biomedical sensor technology continues to evolve, the findings from these studies hold significant promise in revolutionizing medical practices and addressing complex health-related challenges, ultimately leading to better human health and well-being.
